# In Situ Expression of Yak *IL-22* in Mammary Glands as a Treatment for Bovine *Staphylococcus aureus*-Induced Mastitis in Mice

**DOI:** 10.3390/vetsci11100515

**Published:** 2024-10-20

**Authors:** Zening Wang, Daojie Riqing, Liangliang Ma, Mingfeng Jiang, Ciren Zhuoma, Xiaowei Li, Yili Liu

**Affiliations:** 1 College of Animal Science and Veterinary Medicine, Southwest Minzu University, Chengdu 610041, China; wangzn0215@163.com (Z.W.); 19822966564@163.com (D.R.); 22100049@swun.edu.cn (M.J.); 2 College of Grassland Resources, Southwest Minzu University, Chengdu 610041, China; maliangliang2020@163.com; 3 Jiali County Agriculture and Animal Husbandry Science and Technology Service Station, Naqu 852413, China; 19382026835@163.com; 4 Sichuan Longri Livestock Breeding Farm, Hongyuan 624400, China; laoyao1987913@163.com

**Keywords:** *IL-22*, *Staphylococcus aureus*, mastitis, anti-inflammatory, intestinal flora

## Abstract

Since the development of dairy farming, bovine mastitis has been a problem plaguing the whole industry, which has led to a decrease in milk production, a reduction in dairy product quality, and an increase in costs. The use of antibiotics to treat mastitis can cause a series of problems, which can bring a series of harm to the animal itself, such as the development of bacterial resistance and dramatic changes in the gut flora. However, the in vivo and in vitro antibacterial activity of yak Interleukin-22 (IL-22) and its application in mastitis caused by *Staphylococcus aureus* have not been reported. In this study, the mammary gland-specific expression plasmid pLF-IL22 of the yak *IL-22* gene was constructed and expressed in MAC-T cells and mammary tissue of postpartum female mice. The coding region of the *IL-22* gene in yaks is 573 bp, which can encode 190 amino acids, and the homology difference in the *IL-22* gene in yaks is less than 30%, which indicates certain conservation. IL-22 is a hydrophilic protein with a total positive charge of four, the presence of a signal peptide, and the absence of a transmembrane domain. Sufficient expression of *IL-22* effectively inhibited the high expression of inflammatory factors caused by *Staphylococcus aureus*, reduced the symptoms of mammary gland histopathology, and alleviated mastitis. Under the action of IL-22, the intestinal flora of mastitis mice also changed, the abundance of intestinal Bacilli, *Prevotellaceae,* and *Alloprevotella* in mice increased after treatment, and the pathogenic bacteria decreased. These findings provide new insights into the potential application of the yak *IL-22* gene in the treatment of bovine mastitis in the future.

## Simple Summary

Bovine mastitis is one of the most common diseases in farms and has caused huge economic losses to farmers and led to a decline in milk quality. Antibiotics are a common treatment for mastitis. However, due to the emergence of bacterial resistance, they have brought great hidden dangers to public health and safety and also endanger people’s health. Therefore, the development of antibiotic alternatives in the treatment of bovine mastitis is more urgent. Studies have found that yak IL-22 has tissue repair and inflammation inhibition effects. In this study, we used the yak *IL-22* gene as a potential preparation to construct a mammary gland-specific plasmid for gene therapy for bovine mastitis and evaluated the role of this therapy in the disease, laying a foundation for the possible large-scale application of this preparation in the future.

## 1. Introduction

Mastitis is a disease caused by a variety of factors that cause inflammatory reactions in bovine teats or mammary glands. It is one of the most common and frequently occurring diseases in dairy farms. According to statistics, the economic losses caused by mastitis for farms are between CNY 12,000 and 76,000 [1,2]. Mastitis can affect the lactation function of cows, resulting in a decrease in milk yield and nutritional levels and even affecting the reproductive capacity of cows [3]. Studies have found that mastitis is directly related to the pregnancy loss of primiparous cows, and the probability of abortion in cows with clinical mastitis during pregnancy is 2.21 times that in other cows [4]. At present, the treatment of bovine mastitis is mainly based on Western medicine and Chinese herbal medicine, but there are few related literature reports and few reports on other treatment methods [5,6]. The treatment for acute mastitis is still nipple injection of antibiotics, but the long-term use of multiple antibiotics can easily lead to the spectral resistance of pathogenic bacteria and a decline in body immunity, and antibiotics easily remain in milk, so long-term consumption of dairy products with antibiotic residues will lead to resistance in the body, resulting in serious food safety problems [7]. Chinese herbal medicine is widely used in cattle production, but there are still some problems, such as complicated components, complicated operations, and unclear mechanisms of action [8]. To sum up, the prevention and treatment of bovine mastitis lack a set of safe and effective treatment strategies, so it is urgent to develop a new method for the prevention and treatment of bovine mastitis with no chemical residues, no antibiotic residues, low drug resistance, low cost, simple operation, and long-lasting efficacy, which has important theoretical and practical significance for the better development of the dairy industry.

IL-22 is a cytokine secreted by activated T cells, was discovered in 2000 [9]. It acts on target cells mainly by binding to IL-22 receptors selectively expressed on the surfaces of epithelial cells and stromal cells [10]. Once bound to the IL-22 receptor, IL-22 exerts its repair role and can maintain defense mechanisms to inhibit pathogen invasion [11]. IL-22 primarily targets epithelial cells, pancreatic cells, and liver cells in multiple organs, as well as some fibroblast populations [12,13,14,15]. IL-22 also has anti-intestinal infection, anti-liver injury, and other effects. It can promote cell proliferation, prevent apoptosis, and induce acute-phase proteins with anti-inflammatory, antibacterial, and regenerative effects [16,17]. IL-22 improved epithelial dysfunction in both inflammatory bowel disease and *Citrobacter rodentium*-induced colitis models [18]. IL-22 can prevent the invasion of microorganisms and pathogens, enhance whole-body immunity, and improve the production performance of piglets [19]. However, the antimicrobial activity of IL-22 in yaks and its application in *Staphylococcus aureus* (*S. aureus*)-induced mastitis have not been reported. In this study, IL-22 was selected as a candidate drug for the treatment of bovine mastitis, and its anti-inflammatory repair effect was used to treat mammary tissue injured by mastitis. By inserting the *IL-22* gene into the previously constructed mammary tissue-specific expression plasmid, the range of action of IL-22 was limited, drug safety was improved, and *Staphylococcus aureus* infection was significantly reduced in vivo and in vitro.

## 2. Materials and Methods

### 2.1. Bioinformatics Analysis

The sequence homology analysis and phylogenetic tree construction of the yak *IL-22* gene were completed by the Megalign and Mega 7.0 software, respectively. The online software ORF Finder (https://www.ncbi.nlm.nih.gov/orffinder/) “URL (accessed on 2 May 2024)” was used to analyze the yak *IL-22* gene sequences. The basic physicochemical properties of the IL-22 protein in yaks were analyzed by Protparam (https://web.expasy.org/protparam/) “URL (accessed on 2 May 2024)”. The online software Protscale (https://web.expasy.org/protscale/) “URL (accessed on 2 May 2024)” was used to analyze the protein hydrophilicity. The online software TMHMM 2.0 (https://services.healthtech.dtu.dk/services/TMHMM-2.0/) “URL (accessed on 2 May 2024)” and Signalp 4.1 (https://services.healthtech.dtu.dk/services/SignalP-4.1/) “URL (accessed on 2 May 2024)” were used to predict the protein signal peptide sites and transmembrane domains. Through the online software SWISS MODEL (https://swissmodel.expasy.org/)“URL (accessed on 2 May 2024)”, the protein tertiary structure model was constructed.

### 2.2. Vector Construction

The mammary gland-specific expression plasmid pLF-EGFP was constructed and preserved in the laboratory. The plasmid pCMV-IL22 was synthesized by a commercial company (Sangon Biotech, Shanghai, China). The yak *IL-22* gene sequence (XM_005909047) was derived from the National Center for Biotechnology Information (NCBI) database and inserted into a pEGFP-N1 vector with *Kpn* I and *Xba* I restriction sites (TaKaRa). The pLF-IL22 plasmid was constructed by replacing the CMV promoter on pCMV-IL22 with *Vsp* I and *Hind* III (Thermo Fisher, Beijing, China) restriction sites.

### 2.3. Cells and Transfection

MAC-T cells were obtained by the immortalization of bovine primary mammary epithelial cells and cultured in DMEM/F12 medium (Gibco, Waltham, MA, USA) containing 10% heat-inactivated fetal bovine serum (BI, Beijing, China) and 1% penicillin–streptomycin (Biosharp, Beijing, China) [20]. For transient transfection in six-well plates, 1 μg of either pLF-IL22 plasmid was used to transfect the MAC-T cells with Lipofectamine 2000 (Thermo Fisher, Beijing, China). After 48 h, total RNA was extracted from the transfected cells and normal MAC-T cells according to the SteadyPure Universal RNA Extraction Kit (ACCURATE BIOTECHNOLOGY, ChangSha, China). After detecting the purity and concentration of the RNA, it was stored at −80 °C. cDNA was prepared according to the PrimeScript^TM^ FAST RT reagent Kit with a gDNA Eraser (Takara, Beijing, China).

### 2.4. Antimicrobial Tests In Vitro

*S. aureus* was donated by Yangzhou University and isolated from bovine mastitis*. S. aureus* was first streaked on LB solid medium for 16 h at 37 °C, and then single colonies were selected and cultured overnight in LB liquid medium at 37 °C. The concentration of the bacterial suspension was detected by a bacterial turbidity meter, and the bacterial suspension was diluted to the appropriate concentration. Before cell treatment, MAC-T cells were inoculated in a six-well plate at a density of approximately 75% to 80%. The cells were divided into a normal control group (NC), infection group (S. au), positive control group (PC), and pLF-IL22 treatment group (pLF-IL22). The cells in the S. au group were incubated with *S. aureus* for 4 h at 37 °C and 5% CO_2_, and the multiple of infection was 10:1. The cells in the PC group were supplemented with 10% penicillin–streptomycin after being infected with *S. aureus.* After transfection with the pLF-IL22 plasmid for 48 h, the pLF-IL22 group was infected with *S. aureus*. The cells of each group were collected to extract the RNA for reverse transcription.

### 2.5. Animals

The SPF ICR mice selected in this study were 8 weeks old and fed a normal diet and water. The feeding temperature was suitable, and the daily illumination time was about 12 h. The experimental mice were all female mice that were lactating for one week after delivery. The baby mice were isolated in a separate cage 1–2 h before the experiment.

### 2.6. Mastitis Model Test in Mice

A total of 24 ICR mice with similar weights were randomly divided into 4 groups. In the negative control group (NC), six mice were first injected with 50 μL of normal saline and then challenged with 50 μL of normal saline after 24 h. In the infection group (S. au), six mice were only infected with 50 μL of 10^7^ CFU/mL *S. aureus.* In the positive control group (PC), six mice were challenged with 50 μL of 10^7^ CFU/mL *S. aureus* and then infected with 20 μL of lincomycin after 24 h. In the pLF-IL22 group (pLF-IL22), six mice were first injected with 50 μL of normal saline and then challenged with 20 μL of an equal proportion mixture of pLF-IL22 and Pluronic L64 (L64). All injections occurred in the fourth pair of mammary glands on both sides of each mouse. All mice were weighed twice, before the first injection and before execution, and the average value of each group was taken for subsequent data processing. After 48 h, the mice were sacrificed by the cervical dislocation method. The breast samples were quickly obtained under sterile conditions and frozen with liquid nitrogen. HE staining was performed after the breast specimens were made into sections to facilitate subsequent pathological examination.

### 2.7. Quantitative Real-Time Polymerase Chain Reaction (qRT-PCR)

The qRT-PCR primers designed to target *TNF-α*, *IL-6*, *IL-1β*, and *GAPDH* (housekeeping gene) were synthesized by the company Sangon Biotech, Shanghai, China. The LightCycler^®^ 96 System (Roche, Basel, Switzerland) and TB Green^®^ Premix Ex Taq (Takara, Beijing, China) kits were used according to the instructions. The relative expression levels of *TNF-α*, *IL-6*, and *IL-1β* were calculated with the 2^−ΔΔCt^ method [21]. The primers used in the qRT-PCR are shown in Table 1.

### 2.8. Inflammatory Cytokine Enzyme-Linked Immunosorbent Assay

The frozen mouse mammary gland tissue was mixed with precooled normal saline at a ratio of 1:9, and the tissue homogenate was prepared by low-temperature grinding. The supernatant was centrifuged at 12,000× *g* for 15 min, and the inflammatory factors (TNF-α, IL-6, and IL-1β) in the tissue supernatant were detected according to the instructions of the enzyme-linked immunosorbent assay (ELISA) kits (Bioswamp, Wuhan, China).

### 2.9. Pathological Tests

The mammary gland was fixed with paraformaldehyde for 24 h and then dehydrated, embedded, sliced, and dewaxed. The tissue was then subjected to hematoxylin staining for 10–20 min; rinsed in tap water for 1–3 min; differentiated in hydrochloric acid–alcohol for 5–10 s; rinsed in tap water for 1–3 min; put in warm water at 50 °C or a weakly alkaline aqueous solution until it appeared blue; rinsed in tap water for 1–3 min; put in 85% alcohol for 3–5 min; eosin-stained for 3–5 min; washed for 3–5 s; and subjected to gradient alcohol dehydration, transparent xylene, and neutral gum sealing. Finally, the breast tissue was observed under an optical microscope.

### 2.10. Determination of Microbial Diversity

After the intestinal contents of each group were extracted for DNA purification and amplification, the PCR-recovered products were detected and quantified by Qubit^@^ 2.0. PE250 sequencing was performed using the NovaSeq 6000 SP Reagent Kit V1. 5 (Illumina, San Diego, CA, USA). High-quality target sequences for subsequent analysis were assembled and filtered from the original sequencing data. Bioinformatics operations were performed with QIIME2^5^. Amplification, sequencing, and other operations were carried out by Ultra Biotechnology Co., Ltd. (Chendu, China).

### 2.11. Statistics and Data Analysis

The one-factor analysis of variance (ANOVA) test of the experimental data was analyzed using the SPSS 16.0 statistical software. Differences were considered significant at *p* < 0.05 (*). Differences were considered very significant at *p* < 0.01 (**).

## 3. Results

### 3.1. Bioinformatics Analysis of Yak IL-22 Gene

**Figure 1:**
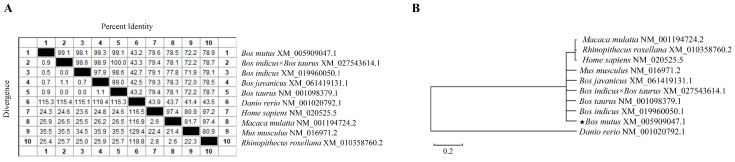
Sequence analysis of yak *IL-22*. (**A**) Homology analysis of yak *IL-22*. (**B**) Phylogenetic tree of yak *IL-22*.

The *IL-22* gene sequence of yaks is the closest to that of *Bos javanicus* (99.3%) (Figure 1A). The CDS region of the yak *IL-22* gene is highly conserved and closely related to that of *Bos indicus*, *Bos taurus*, *Bos indicus* × *Bos taurus*, *Bos javanicus*, and *Mus musculus* (Figure 1B).

**Figure 2:**
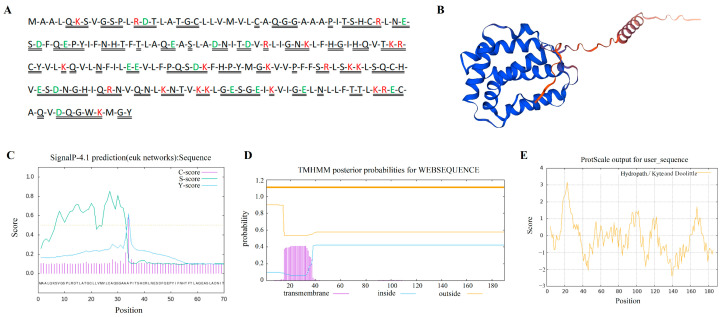
Molecular characteristics of yak IL-22 protein. (**A**) Amino acid sequence of IL-22 protein. Red text letters represent positively charged amino acids; green represents negatively charged amino acids; double solid lines represent hydrophobic residues. (**B**) Three-dimensional structure of IL-22 protein. (**C**) Prediction of IL-22 protein signaling peptide. (**D**) Prediction of IL-22 protein transmembrane structure. (**E**) Prediction of hydrophilicity of IL-22 protein.

The yak *IL-22* gene has an open reading frame length of 573 bp and encodes 190 amino acids, of which leucine (Leu) is the most abundant (Figure 2A). The 3D structure prediction of the IL-22 protein is shown in Figure 2B. Its theoretical isoelectric point is 8.73, its molecular weight is 21.35 kD, and the protein has four positive charges. Its molecular formula is C_953_H_1519_N_263_O_273_S_10_, its total number of atoms is 3018, its aliphatic index is 94.37, its instability coefficient is 41.61, and it is an unstable protein with a half-life of 30 h. The mean total hydrophilicity of the IL-22 protein is −0.124. The prediction results show that the transmembrane helical region is not included in the yak IL-22 protein sequence and contains a signal peptide (Figure 2C,D). The results of the ProtScale analysis and physicochemical property analysis show that the yak IL-22 protein is a hydrophilic protein (Figure 2E).

### 3.2. The Conversion of Inflammatory Factors In Vivo and In Vitro

**Figure 3:**
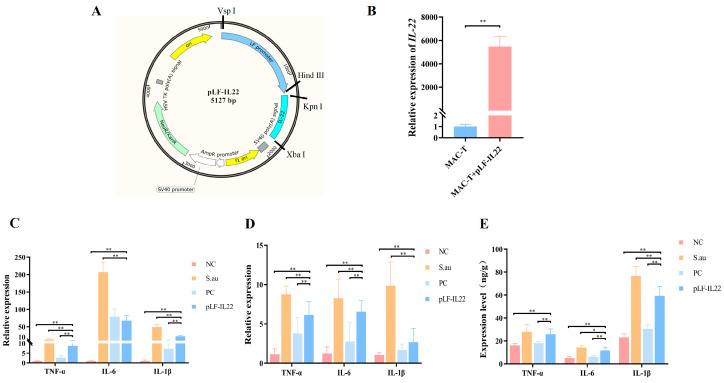
Construction and antibacterial effect of pLF-IL22. (**A**) Plasmid construction map of pLF-IL22. (**B**) Overexpression of pLF-IL22 plasmid in MAC-T cells (*n* = 3). (**C**) RT-qPCR test of inflammatory factors expression in cell therapy (*n* = 3). (**D**) RT-qPCR test of inflammatory factors expression in the treatment of mastitis in mice (*n* = 6). (**E**) Protein contents of inflammatory cytokines in serum of mice (*n* = 6). Differences were considered significant at *p* < 0.05 (*). Differences were considered very significant at *p* < 0.01 (**).

The sequence characteristics of the pLF-IL22 plasmid are shown in Figure 3A. The RNA quality was qualified, the concentration was greater than 100 ng/μL, the OD260/OD280 was 1.8–2.4, and the OD260/OD230 was 1.5–2.4. It is obvious that the relative expression of *IL-22* in cells was significantly increased after transfection (*p* < 0.01) (Figure 3B). Under the invasion of *S. aureus*, the relative expression of three inflammatory factors in the S. au group was significantly increased compared with that in the NC group (*p* < 0.01). However, with the expression of *IL-22*, a very significant decrease in the inflammatory factors was observed in the transfected cells compared with the S. au group (*p* < 0.01) (Figure 3C). By detecting the relative expression levels of the inflammatory factors in mouse mammary gland tissues (Figure 3D), it was found that the expression levels of the three inflammatory factors in the pLF-IL22 group were significantly lower than those in the S. au group (*p* < 0.01). The results of the ELISA showed that the protein contents of TNF-α, IL-6, and IL-1β in the serum of the pLF-IL22 group were decreased to different degrees after treatment. The decrease in IL-1β was the most obvious (*p* < 0.01), followed by IL-6 (*p* < 0.05), and the decrease in TNF-α was the lowest (Figure 3E).

### 3.3. Body Weight Changes of Mice

**Figure 4:**
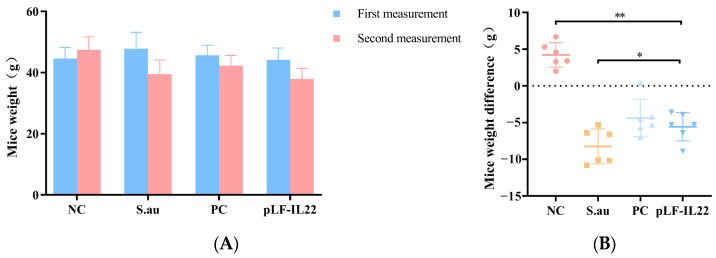
Body weight changes of mice (*n* = 6). (**A**) Body weights of mice in each group at the time of two injections. (**B**) Weight differences of mice in each group. Differences were considered significant at *p* < 0.05 (*). Differences were considered very significant at *p* < 0.01 (**).

The body weight of mice may be related to the development of the disease. The initial body weight of all mice was not significantly different, but the body weight of the mice in each group changed significantly after treatment (Figure 4A). Except for the NC group, the weights of the other three groups of mice were reduced. The body weight of the S. au group decreased significantly, followed by the pLF-IL22 group and the PC group (Figure 4B).

### 3.4. Histopathological Changes

**Figure 5:**
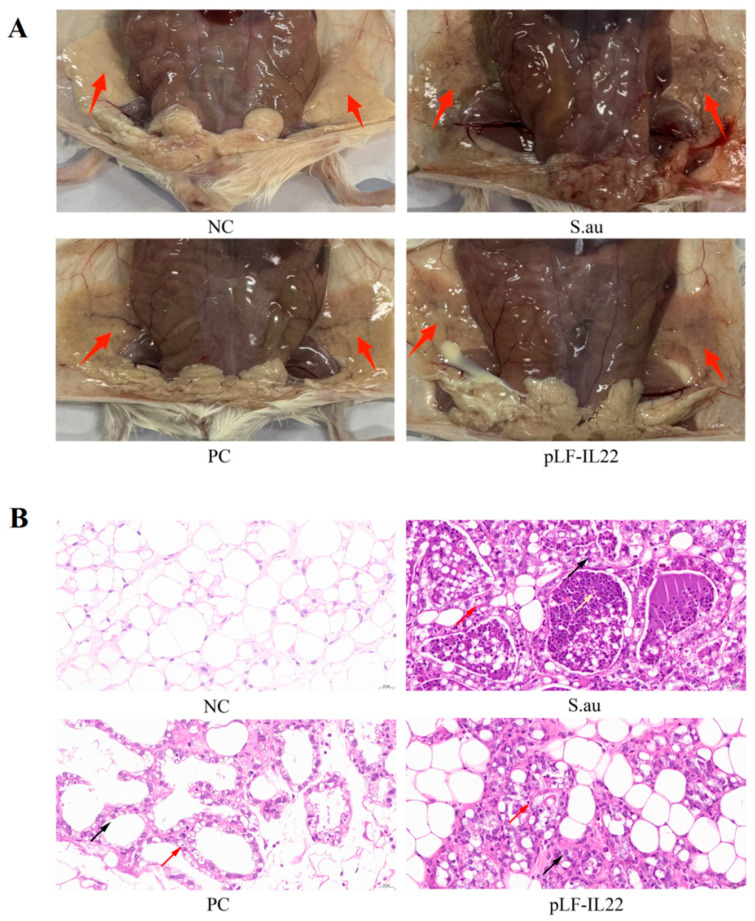
Pathology and anatomy of mammary glands in mice. (**A**) Anatomical observation of mammary glands in mice of each group. Red arrows indicate the mammary glands in mice. (**B**) Pathological observation of mammary glands in mice of each group. Red arrows indicate epithelial cell necrosis, black arrows indicate epithelial cell degeneration, and yellow arrows indicate secretions and inflammatory cells.

*S. aureus* infection can lead to mammary gland lesions. In the S. au group, the trunks of the mice were thin and small, and there were obvious bleeding points in the mammary glands. There was a certain degree of mammary gland swelling and erosion, which was very different from the NC group. The PC group and pLF-IL22 groups showed mild inflammatory symptoms, such as dry and red mammary glands (Figure 5A). The breast paraffin sections intuitively clarified the therapeutic effect. No obvious lesions were found in the NC group. After *S. aureus* infection, acinar expansion, acinar epithelial cell degeneration (black arrow), and glandular epithelial cells with occasional necrosis (red arrow) were observed. The glandular cavity was filled with secretions and inflammatory cells (yellow arrow). The inflammatory response was alleviated, the degeneration and necrosis of epithelial cells were reduced, and the degree of inflammatory cell infiltration was low 48 h after plasmid injection (Figure 5B).

### 3.5. Effects on Composition of Intestinal Flora

**Figure 6:**
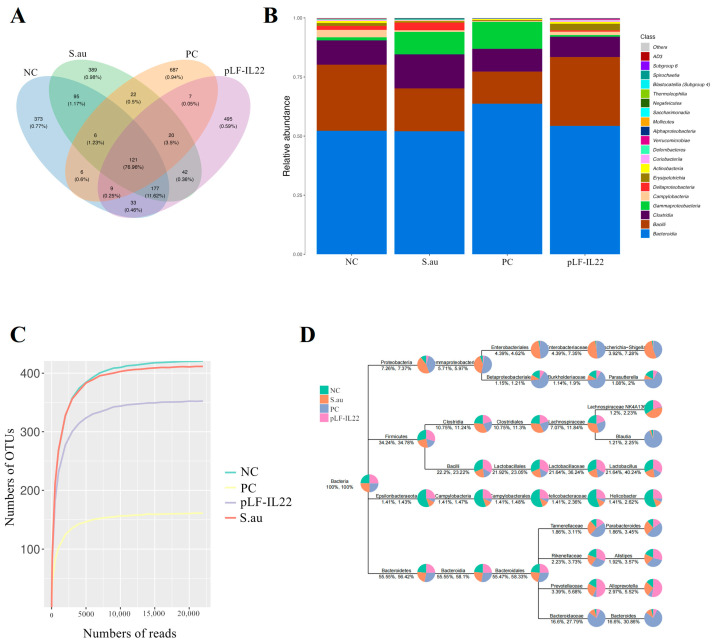
Abundance of gut microbiota in mice (*n* = 6)**.** (**A**) The Venn diagram was made according to the OTU abundance table, and the existence of OTUs in each sample group was used to count each set. (**B**) Histogram of relative abundance of species at the class level. (**C**) Sample dilution curve. (**D**) The top 10 genera with the highest relative abundances were screened for species classification tree statistics.

Each operational taxonomic unit (OTU) is usually considered a microbial species. The numbers of OTUs in the NC group, S. au group, PC group, and pLF-IL22 group were 820, 872, 878, and 904, and the numbers of unique OTUs were 373, 389, 687, and 495, respectively. The number of total OTUs was 121 (Figure 6A). At the class level, pLF-IL22 increased the relative abundance of Bacille, and Gammaproteobacteria decreased compared with the NC group and S. au group. pLF-IL22 increased the relative abundance of Lachnospiraceae and Bacteroidia, and Clostridia and Bacteroidanceae decreased compared with the NC group and S. au group (Figure 6B). Lincomycin induced the greatest changes in the intestinal flora. The species classification tree statistics reflect the top 10 genera with the highest relative abundances (Figure 6D). The sample dilution curve reflects the sequencing depth of microorganisms (Figure 6C), and the flat curve indicates that the number of species in this environment did not increase significantly with sequencing [22].

### 3.6. Differences in Intestinal Flora and Prediction of Flora Function

**Figure 7:**
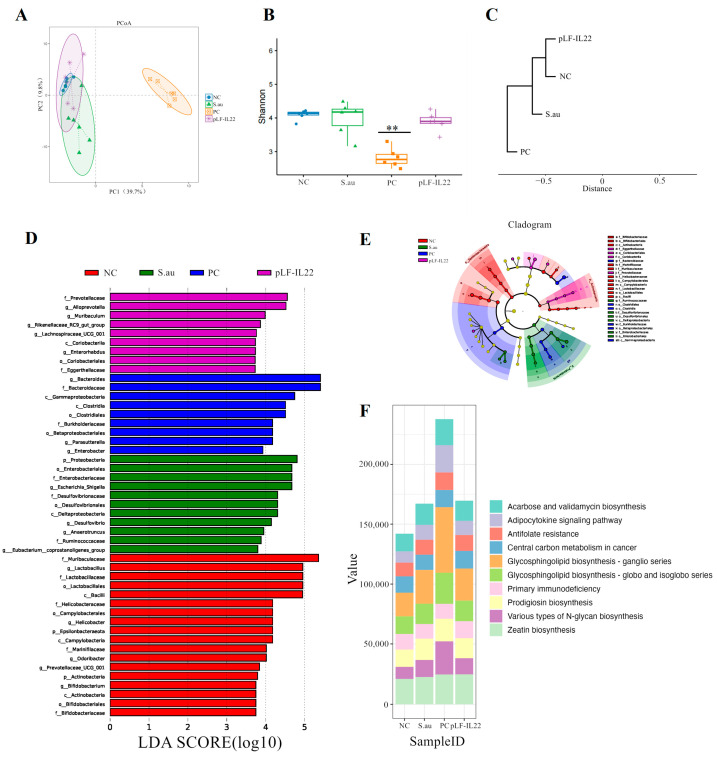
Intestinal microbial diversity, LEfSe, and function prediction (*n* = 6). (**A**) PCoA score plot based on OTU level. (**B**) Shannon diversity index of intestinal bacteria among groups. Differences were considered very significant at *p* < 0.01 (**). (**C**) UPGMA clustering tree based on weighted UniFrac distance. (**D**) Linear discriminant analysis (LDA). (**E**) Results of taxonomic cladograms. (**F**) Prediction of intestinal flora function.

β-diversity analysis showed that there was no significant difference between the NC group, S. au group, and pLF-IL22 group, but there were significant differences in the structure and composition of the intestinal bacterial community in the PC group (Figure 7A). The Shannon index was used to measure the species diversity. As shown in Figure 7B, the pLF-IL22 group did not significantly change the intestinal colonies of mice, and even the S. au group did not show significant differences, but the changes in the intestinal tract of mice by lincomycin were significant. The results of the cluster analysis show that the pLF-IL22 group was first clustered with the NC group, followed by the S. au group, and, finally, the PC group (Figure 7C). Linear discriminant analysis (LDA) and taxonomic cladograms effect size (LEfSe) analysis can find important and significantly different species between groups (Figure 7D). In the pLF-IL22 group, we found *Prevotellaceae* enrichment at the family level and *Alloprevotella* enrichment at the genus level. The enrichment of *Bacteroides* at the family and genus levels, Gammaproteobacteria at the class level, *Clostridiales* at the class and order levels, *Burkholderiaceae* at the family level, *Betaproteobacteriales* at the order level, *Parasutterella* at the genus level were observed in the PC group. In the S. au group, we found Proteobacteria enrichment at the phylum level, *Enterobacteriaceae* enrichment at the order and family levels, *Escherichia-shigella* enrichment at the genus level, *Desulfovibrio* at the order, family, and genus levels, and Deltaproteobacteria enrichment at the class level. In addition, in the NC group, we observed the enrichment of *Muribaculaceae* at the family level, *Lactobacillus* at the order, family, and genus levels, Bacilli at the class level, *Helicobacter* at the family and genus levels, *Campylobacterales* at the class and order levels, and Epsilonbacteraeota at the phylum level. The results of the taxonomic cladograms are basically the same (Figure 7E).

PICRUSt2 can be used to predict microbial community function based on amplicon sequencing results combined with a genome database. The results of the colony function prediction for the different treatment groups mainly focused on zeatin biosynthesis, various types of N-glycan biosynthesis, prodigiosin biosynthesis, primary immunodeficiency, glycosphingolipid biosynthesis (globo and isoglobo series), glycosphingolipid biosynthesis (ganglio series), central carbon metabolism in cancer, antifolate resistance, the adipocytokine signaling pathway, and acarbose and validamycin biosynthesis (Figure 7F).

## 4. Discussion

Mastitis is a high-incidence disease in large-scale dairy farms and has a serious impact on the health and welfare of dairy cows [23]. Broad-spectrum antibiotics are commonly used in the prevention and treatment of mastitis, but the generation of drug-resistant strains, the decrease in therapeutic effects, and the solution of drug residues in milk are urgent [24]. We developed a targeted mastitis gene therapy protocol using the yak *IL-22* gene as an anti-inflammatory agent instead of antibiotics to suppress mastitis caused by *S. aureus*. Some studies have found that the similarity of the *IL-22* gene between mice and humans is only 78.2% [25]. In this study, the homology of the *IL-22* gene between yaks and other cattle breeds was 98%, but the sequence similarity between yaks and other mammals such as *Home sapiens*, *Mus musculus*, *Macaca mulatta*, and *Rhinopithecus rexellana* was only about 70%. This discovery made it simpler to apply the yak *IL-22* gene to bovine mastitis. IL-22 belongs to the IL-10 family, which also includes IL-10, IL-19, IL-20, IL-24, and IL-26. The structure of the IL-22 protein in yaks is similar to other members of the IL-10 family, with a bundle structure and more α-helixes [26]. The yak IL-22 protein has a signaling peptide, and the protein may be extracellular to play its role. IL-22 does not directly regulate immune cell function but plays a protective role by inducing target cells to produce antibacterial proteins and specific chemokines [27]. The yak IL-22 protein may have the potential to protect bovine mammary tissue from *S. aureus* invasion.

IL-22 regulates tissue responses, especially in inflammatory environments. It is highly upregulated in patients with many chronic inflammatory diseases, such as psoriasis, periodontitis, rheumatoid arthritis, and inflammatory bowel disease (IBD) [27,28,29,30]. Yak IL-22 may play a similar role in bovine mastitis [31]. In this experiment, the expression of the pLF-IL22 plasmid greatly reduced the increase in inflammatory factors caused by *S. aureus* infection in MAC-T cells and had a certain easing effect on inflammation. Studies have shown that IL-22 can reduce the inflammatory response in colon and lipopolysaccharide-induced acute liver injury (ALI), and its elevation effectively inhibits the expression of the inflammatory cytokines IL-1β and IL-6, while the effect is opposite after knockdown [32,33]. In mice, the expression of the yak *IL-22* gene also inhibited the expression of inflammatory factors, but the inhibitory effect was decreased compared with that in cells. This may be due to the relatively low homology of the *IL-22* gene between yaks and mice, which complicates the translation of yak *IL-22* genes into mouse diseases, or it may be because of the presence of the IL-22-binding protein (IL-22BP) [34]. Dendritic cells (DCs) can produce high levels of IL-22BP, which binds to IL-22 with a higher affinity than transmembrane receptors, which may result in some IL-22 having no biological activity [35]. The weight of mice is regulated by many factors [36]. There may be a certain relationship between the weight of mice and inflammation. In this experiment, we found an interesting phenomenon, where the weight change of mice was positively correlated with the development of mastitis, that is, the more severe the infection of *S. aureus*, the more obvious the weight loss of mice, while the expression of the *IL-22* gene in yaks inhibited weight loss. *IL-22* plays an important role in epithelial regeneration, and its expression is related to the expression of tight-junction proteins and pathogen-protective factors on the cell surface, which contributes to the regeneration of the endometrial layer in the inflammatory environment [37]. The pathological results show that IL-22 had a certain protective effect on the mammary tissue. Compared with the PC group, the mammary tissue structure was more complete, and the inflammatory symptoms were relieved compared with the S. au group.

Normal intestinal flora can regulate the permeability of intestinal epithelial cells, stimulate substance metabolism and the immune response, and keep the intestinal microenvironment in a stable state for a long time. At present, changes in the intestinal flora are mostly observed in intestinal diseases, while little attention is paid to changes in the intestinal flora caused by other diseases, especially mastitis. We conducted intestinal flora analysis on the mice in the NC group, S. au group, PC group, and pLF-IL22 group to explore the effects of mastitis and its treatment on the intestinal flora. We found that pLF-IL22 treatment and even *S. aureus* mammary infection had no significant effect on the intestinal flora, while lincomycin induced obvious changes in the intestinal flora. Antibiotics increased the number of *Bacteroides*, which belonged to Bacteroidia, and the proportion of *Bacteroides* was relatively stable in the intestinal tract. The disorder may carry the risk of disease [38]. Bacilli is a class of Firmicutes, most of which are beneficial bacteria, such as *Lactobacillus* and *Ruminococcus* [39]. They can produce acetate, butyrate, lactate, and antibacterial substances to prevent pathogens from interfering with health [40,41]. Compared with the NC group, the Bacilli quantity increased in the pLF-IL22 group but decreased in the S. au and PC groups. The cluster comparison of various methods showed that the colony structure of the pLF-IL22 group was more similar to that of the NC group, indicating that the treatment with pLF-IL22 did not cause additional damage to the intestinal flora of mice, while the treatment with lincomycin caused significant changes in the intestinal flora and a reduction in beneficial bacteria. pLF-IL22 offers a safer treatment option with less impact on animals. *Prevotellaceae* usually plays the role of “probiotics”, and its reduction is associated with certain diseases [42]. *Prevotella histicola* alone or in combination can effectively reduce the pro-inflammatory response associated with multiple sclerosis and improve the disease [43,44]. The abundance of *Alloprevotella* is negatively correlated with obesity, diabetes, and metabolic syndrome [45]. *Prevotellaceae* and *Alloprevotella* were enriched in the pLF-IL22 group*. Proteobacteria*, *Enterobacteriaceae*, *Escherichia-Shigella*, and other pathogenic bacteria were enriched in the S. au group [46,47,48]. This also shows that pLF-IL22 has a positive effect on the enrichment of beneficial bacteria in intestinal microorganisms and has a potential therapeutic effect. The results of the colony function prediction showed that these functions were mainly enriched in translation, repair, nucleotide metabolism, and immune diseases and also reflected the enrichment effect of pLF-IL22 on probiotics in the intestine.

## 5. Conclusions

In conclusion, this study demonstrates the potential of the pLF-IL22 plasmid as a mastitis treatment. The yak *IL-22* gene is highly conserved in cattle and can be used as a widespread mastitis therapy gene. Moreover, yak IL-22 showed a good therapeutic effect in MAC-T cells and mice, reducing the damage caused by *S. aureus*. This indicates that the yak IL-22 protein, as an internal cytokine, has few toxic side effects and can treat mastitis safely and effectively. The application of the yak *IL-22* gene in the treatment of bovine mastitis in actual production still needs to be further developed, and the improvement of the biological activity of the IL-22 protein in vivo in gene therapy to achieve better therapeutic effects also needs to be further studied.

## Figures and Tables

**Table 1:** Primer sequences.

Gene	Size	Primer Sequence (5′-3′)
*GAPDH*-F-Cell	208 bp	TGTTGTGGATCTGACCTGCC
*GAPDH*-R-Cell		AAGTCGCAGGAGACAACCTG
*GAPDH*-F-Mice	130 bp	AGGTCGGTGTGAACGGATTTG
*GAPDH*-R-Mice		GGGGTCGTTGATGGCAACA
*TNF-α*-F-Cell	126 bp	GTTCTCCCCATGACACCACCTG
*TNF-α*-R-Cell		GGGAGAAGAGAGTCAGACAGGC
*IL-6*-F-Cell	134 bp	ACAAGCGCCTTCACTCCATT
*IL-6*-R-Cell		AGAAGTAGTCTGCCTGGGGT
*IL-1β*-F-Cell	140 bp	ATGGCAACCGTACCTGAACC
*IL-1β*-R-Cell		CCATCTCCCATGGAACCGAG
*TNF-α*-F-Mice	126 bp	CCACCACGCTCTTCTGTCTA
*TNF-α*-R-Mice		CCACTTGGTGGTTTGTGAGTG
*IL-6*-F-Mice	138 bp	GGTCTTCTGGAGTACCATAGC
*IL-6*-R-Mice		GTGACTCCAGCTTATCTCTTGGT
*IL-1β*-F-Mice	136 bp	TGCCACCTTTTGACAGTGATG
*IL-1β*-R-Mice		ATGTGCTGCTGCGAGATTTG

## Data Availability

The data presented in this study are contained within this article.
